# Atypical Presentation of a Type A Aortic Dissection in a Patient With an Undiagnosed Genetic Predisposition

**DOI:** 10.7759/cureus.56394

**Published:** 2024-03-18

**Authors:** Nishal N Patel, Adam Kurnick, Inna Bukharovich

**Affiliations:** 1 Department of Medicine, State University of New York Downstate Health Sciences University, Brooklyn, USA; 2 Department of Cardiology, State University of New York Downstate Health Sciences University, Brooklyn, USA

**Keywords:** atypical chest pain, aortic injury, aortic surgery, atypical presentation of aortic dissection, type a aortic dissection

## Abstract

A 60-year-old female with a past medical history of hypertension presents to the ED with one day of throbbing left knee pain with associated numbness that worsened with ambulation. EKG shows lateral T-wave inversions with no prior for comparison. The patient had bloodwork drawn and a chest x-ray ordered. Her pain was improving with acetaminophen, and during further workup, she went into cardiac arrest. The advanced cardiac life support protocol was initiated, the patient was intubated, and point-of-care ultrasound revealed pericardial effusion. Despite all her efforts, she couldn’t regain consciousness and was pronounced dead. An autopsy confirmed that the patient suffered a type A aortic dissection (AD), with findings indicating a predisposing genetic component. This case confirms that type A AD can present with different clinical symptoms and that a high index of suspicion is crucial in providing lifesaving measures.

## Introduction

Aortic dissection (AD) is a critical and potentially life-threatening condition characterized by the separation of the layers of the aortic wall, creating a false lumen through which blood can flow. This condition demands immediate medical attention and surgical intervention due to its high mortality rate if left untreated. AD is relatively rare, with an estimated incidence of 2.9 cases per 100,000 person-years, but its consequences can be devastating if not promptly diagnosed and managed [1]. The clinical presentation of AD can be highly variable, often mimicking other cardiovascular conditions such as myocardial infarction or pulmonary embolism. Common symptoms include severe, sudden-onset chest pain that is often described as tearing or ripping in nature and radiating to the back or neck. Patients may also experience symptoms related to end-organ ischemia, including neurological deficits, limb weakness, or abdominal pain [2]. We report the case of an AD patient who presented with the primary symptom of lower limb pain. The importance of this case is to highlight the non-stereotypical features of AD in order to provide rapid, life-saving treatment.

## Case presentation

**Figure 1:**
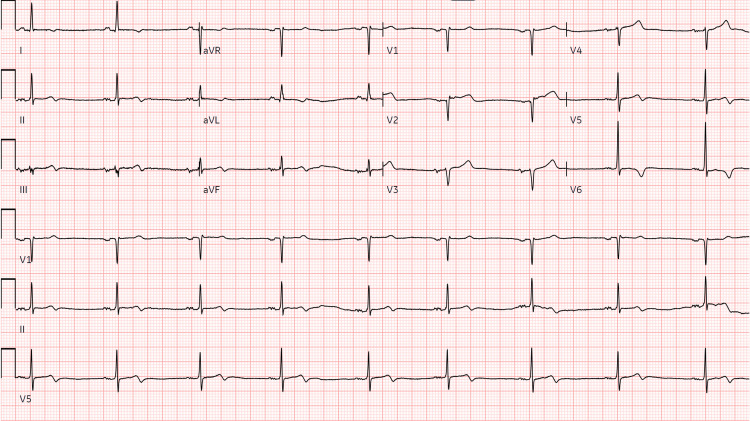
EKG on presentation showing sinus bradycardia, anterolateral ischemia (age undetermined), and inverted T-waves on lateral leads EKG: electrocardiogram

A 60-year-old female with a past medical history of hypertension presents to the ED complaining of left-leg throbbing pain and subjective numbness around her left knee. A systemic review revealed pressure-like central chest pain and intermittent cramping of the left leg aggravated by walking. On presentation, the vital signs were as follows: the blood pressure was ~122/65, the pulse was ~57, and the EKG showed sinus bradycardia with lateral T-wave inversion, a sign of myocardial ischemia (Figure 1).

Physical exam findings were positive for bradycardia, decreased dorsalis pedis pulse in the left lower extremity (LLE) compared to the right lower extremity, and subjectively decreased LLE sensation circumferentially from the lower one-third of the shin down to the foot. Bilateral lower extremity strength was intact. No mottling or discoloration of the lower extremities was observed. Labs revealed mild leukocytosis, elevated creatinine without a known baseline, and normal troponin T (Table 1).

**Table 1:** Laboratory findings

Laboratory values	Result	Reference value
Blood urea nitrogen	26.0	8.0-23.0 mg/dL
Creatinine	1.43	0.5-0.9 mg/dL
Estimated glomerular filtration rate	42.0	≥60 ml/min/1.73m2
Carbon dioxide	20	24-31 mmol/L
Glucose	134	70-99 mg/dL
Troponin T	0.010	≤ to 0.010 ng/mL
White cell count	13.03	4.50-10.90 K/μL
Absolute neutrophil count	11.74	2.5-7.0 K/μL
Neutrophil percentage	90.1	40-60%
Hemoglobin	12.6	12.0-16.0 g/dL
Mean corpuscular volume	95.2	78.0-95.0 fL

Repeat vitals remained stable without significant change. The patient’s pain improved after the administration of acetaminophen 650 mg, and during further workup, the patient suddenly went into cardiac arrest. The patient was brought over to the critical care trauma bay with cardiopulmonary resuscitation (CPR) in progress. The rhythm was pulseless electrical activity; an advanced cardiac life support protocol was initiated; and the patient was promptly intubated. Initial point-of-care ultrasound (POCUS) showed good lung sliding and no pericardial effusion. A tissue plasminogen activator was then given due to suspicion of massive pulmonary embolism. Repeat POCUS showed a newly developed pericardial effusion, and drainage was attempted. Despite all efforts, she couldn’t obtain a return of spontaneous circulation and was pronounced dead. An unrestricted autopsy request was signed by the proper next of kin. On autopsy gross examination, the heart weighed ~380 grams (270-360 grams) with a dark red appearance on the pericardial surface and 100 ml of hemopericardium. The aortic arch showed intramural hematoma and the presence of a true lumen and a false lumen of the aorta. Autopsy also noted aneurysms in the abdominal aorta and in the brain's circle of Willis, as well as the vascular pathology of tortuous coronary artery and pulmonary artery branches, suggesting an underlying genetic cause of this patient's vascular pathology.

## Discussion

AD primarily affects individuals aged 50 and older, with a slight male predominance [3]. While the incidence is relatively low, the condition remains a significant contributor to cardiovascular morbidity and mortality, accounting for approximately 1-2% of all aortic-related deaths [4]. AD is often associated with several predisposing factors, including hypertension, atherosclerosis, connective tissue disorders (e.g., Marfan syndrome, Ehlers-Danlos syndrome), bicuspid aortic valve, prior aortic surgery, trauma, and cocaine use [5]. Among these, hypertension is the most common risk factor, present in approximately 70-80% of patients diagnosed with AD [6].

AD can also occur in patients with undiagnosed genetic syndromes. Approximately 20% of thoracic aneurysm aortic dissection (TAAD) patients without a known genetic syndrome have a family history of TAAD, indicating a significant genetic component to this disease [7]. At least 37 genes have been identified in association with TAAD. Given the possibility of an inheritable disease, it is recommended that all first-degree relatives of an individual with TAAD be evaluated by echocardiogram, CT scan, and ultrasound in order to completely evaluate the aorta. The children of the patient should be evaluated 10 years before the age of the dissection or at age 50, whichever is younger.

The clinical presentation of AD can be highly variable, often mimicking other cardiovascular conditions such as myocardial infarction or pulmonary embolism. Common symptoms include severe, sudden-onset chest pain that is often described as tearing or ripping in nature, radiating to the back or neck. Patients may also experience symptoms related to end-organ ischemia, including neurological deficits, limb weakness, or abdominal pain [2].

Timely management of AD is essential to reducing mortality. Treatment often involves surgical intervention to repair or replace the affected aortic segment. The choice of intervention depends on the type and location of the dissection, patient comorbidities, and surgical expertise [8].

Mechanical stress on the aorta, which can be caused by compressions during resuscitation, can also be a risk factor for dissection. Excessive force applied during CPR can lead to elevated intraluminal pressure, especially in the aortic arch. This heightened pressure can potentially worsen an existing weakness in the aortic wall or create conditions conducive to the initiation of a dissection. Individuals with underlying connective tissue disorders, hypertension, or other predisposing factors are particularly vulnerable to such mechanical stress [9].

## Conclusions

AD is a rare but potentially fatal medical emergency that requires prompt recognition and intervention. The genetic components of this condition often go undiagnosed, so it is essential for patients to obtain and share their family medical history with their providers for prompt evaluation and screening. AD can present with signs that deviate from the classic clinical presentation of sudden, severe chest or back pain. A thorough understanding of the condition, predisposing risk factors, and the available diagnostic and treatment options is essential for healthcare providers to ensure optimal patient outcomes.
